# Immunoexpression of α2-integrin and Hsp47 in hereditary gingival fibromatosis and gingival fibromatosis-associated dental abnormalities

**DOI:** 10.4317/medoral.17970

**Published:** 2012-12-10

**Authors:** João R. Vieira-Júnior, Carolina de Oliveira-Santos, Ricardo Della-Coletta, Daiane Cristianismo-Costa, Lívia M.-R. Paranaíba, Hercílio Martelli-Júnior

**Affiliations:** 1 Dental School, State University of Montes Claros, Unimontes, Montes Claros, Minas Gerais State, Brazil; 2 Dental School, State University of Campinas, Unicamp, Piracicaba, São Paulo State, Brazil

## Abstract

Objective: The purpose of the present study was to investigate the expression of the α2-integrin subunit and heat shock protein 47 (Hsp47) in two families with isolated gingival fibromatosis (GF) form and one family with GF associated with dental abnormalities and normal gingiva (NG). Study Design: Immunohistochemistry was performed with antibodies against α2-integrin and Hsp47 in specimens from two unrelated families with hereditary gingival fibromatosis (Families 1 and 2) and from one family with a gingival fibromatosis-associated dental abnormality (Family 3); NG samples were used for comparison. The results were analysed statistically. Results: Immunoreactivity for α2-integrin and Hsp47 was observed in the nucleus of epithelial cells of both the basal and suprabasal layer and a more discreet signal was noted in connective tissue in all study samples. Hsp47 showed higher immunoreactivity in Family 2 compared with the other families (p≤0.05). Despite the markup α2-integrin was higher in Family 3 there was no statistically significant difference between the families studied (p≥0.05). Conclusions: Our results confirmed the heterogeneity of GF, such that similar patterns of expression of the condition may show differences in the expression of proteins such as Hsp47. Although no difference in α2-integrin expression was observed between GF and NG groups, future studies are necessary to determine the exact role of this protein in the various forms of GF and whether it contributes to GF pathogenesis.

** Key words:**Gingival fibromatosis, integrin alpha2, heat shock protein Hsp47.

## Introduction

Gingival fibromatosis (GF) is the overgrowth of the gingiva characterized by the expansion and accumulation of the connective tissue with the occasional presence of an increased number of cells (1). It is hereditary or is induced as a side effect of systemic drugs including the phenytoin, cyclosporin, and the nifedipine (2,3). The hereditary form is a rare oral condition manifested by a slowly progressive, benign, localized or generalized enlargement of the gingiva (4) with incidence of 1:750,000 live births (3,5), in which case it is referred to as hereditary gingival fibromatosis (HGF) (3). HGF was recently reported in association with dental abnormalities (DA) and in this case, thin generalized hypoplastic amelogenesis imperfecta was indicated as the main dental feature (6).

 Integrins are a large family of heterodimeric membrane glycoproteins that play important roles in numerous cellular processes involving cell–ECM and cell–cell interactions. It has been established that α1β1 and α2β1 regulate collagen and MMP-1 gene expression, where the former reduces the expression of collagen upon ligand binding and the latter stimulates collagen and colla-genase gene expression in both two-and three-dimensional cultures (7-9). Collagen-binding integrins were reported to play a crucial role in maintaining the structural and mechanical properties of the collagen matrix in skin tissues (10). Although excess ECM accumulation seems to be a common feature of HGF, few studies have investigated the role of integrins in this condition.

Heat shock protein (Hsp) 47 is another resident ER protein, first identified as the major collagen-binding heat-inducible glycoprotein in fibroblasts. It is characterized by the correlation of its expression with that of various types of collagen in various tissues and under some pathophysiological conditions (11).

Hsp47 is involved in several fibrotic diseases such as connective tissue diseases and dermal fibrotic diseases. The aim of this study was to determine the expression of the α2-integrin and Hsp47 proteins in two distinct families affected by an isolated form of GF and one family affected by GF and DA syndrome.

## Material and Methods

-Tissue samples

This cross-sectional study included gingival samples from members of two families with HGF and one family with GF and DA syndrome. Clinical, histological and genetic data from these three families have been reported previously. Four tissue samples were selected from the first HGF family (12) (designated GF Family 1 for the purposes of this study), along with four from the second HGF family (13) (designated GF Family 2) and four from the family affected by the GF and DA syndrome (6) (designated GF Family 3). Four samples of normal gingiva (NG) were used as controls. New sections from paraffin-embedded blocks were stained with haematoxylin and eosin (H&E) or used for immunohistochemical analysis. This study was carried out in accordance with the World Medical Association Declaration of Helsinki and with the approval of the Human Research Ethics Committee of the University. All patients gave consent to participate in the study.

-Immunohistochemical analysis

Immunohistochemistry was performed using the streptavidin-biotin method. Briefly, after de-waxing and hydration in graded alcohol solutions, sections were treated with 3% H2O2, followed by incubation with 10 mM citric acid pH 6.0 in an electric pressure cooker to allow for antigen retrieval. After washing with phosphate-buffered saline (PBS), sections were incubated with primary antibodies (antihuman α2-integrin, diluted 1:50, Santa Cruz Biotechnology, CA, USA; antihuman Hsp47, diluted 1:50, Santa Cruz Biotechnology, CA, USA). Following incubation with α2-integrin and Hsp47 antibody, slides were washed in PBS and incubated with the secondary antibody (StreptABComplex/ HRP, Dako Corp., Carpenteria, CA, USA, diluted 1:20) for 30 min at 37 °C. Slides were then incubated with the LSAB system (Labeled Streptavidin Biotin, Dako, diluted 1:100) for 30 min at 37 °C. Reactions were developed by incubating the sections with 0.6 mg/ml 3,39-diaminobenzidine tetrahydrochloride (Sigma, St. Louis, MO, USA) containing 0.01% H2O2. Negative and positive controls were performed for each antibody.

For immunohistochemical assessment, a reproductible semi-quantitative analysis was performed as previously described (14). After scanning and examination of the entire section, five representative areas were evaluated with high power. Scoring of the immunohistochemical results were performed by two authors, whose were blinded with regard to the clinical data. At each selected field, epithelial cells were classified with respect to staining intensity ranging from 1 to 4, and the percentage of positive cells was estimated with the aid of an image analyzer (Nikon NIS-Elements-2.35, Nikon Corporation, Melville, USA). The expression value for each field was then calculated by multiplying the percentage of positive cells by the numerical value of that intensity. For statistical analysis the mean of each sample was used.

-Statistical analysis

Data are presented as the mean±SD for each group. The Kruskal-Wallis multiple comparison test was used to test group effects and Spearman’s correlation test was performed to determine the correlations between immunohistochemical markers. In our comparisons, p≤0.05 was considered indicative of statistical significance.

## Results

**Figure 1:**
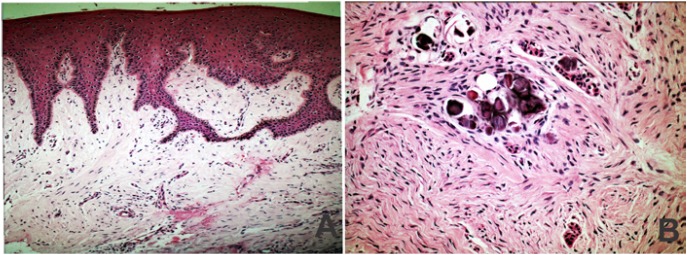
Histological features of the different GF forms in this study. Shown here are representative samples of GF Family 1 and GF Family 2 A), and GF Family 3 B). GF samples revealed a well-structured epithelium with elongated and thin papillae inserted into deep fibrous connective tissue with collagen fibre bundles running in all directions. Additionally, GF tissues from Family 3 demonstrated calcifications and odontogenic epithelial rests in the dense fibrous connective tissue (original magnification x200).

Histological examination of samples of gingival tissue from both GF Families 1 and 2 by HE staining revealed similar characteristics. The gingival tissues included a well-structured epithelium with elongated and thin papillae inserted into deep fibrous connective tissue with collagen fibre bundles running in all directions (Fig. 1). In GF Family 3, a large number of cementicles and odontogenic epithelium rests were observed in the connective tissue in addition to the previously mentioned features (Fig. 1).

**Figure 2:**
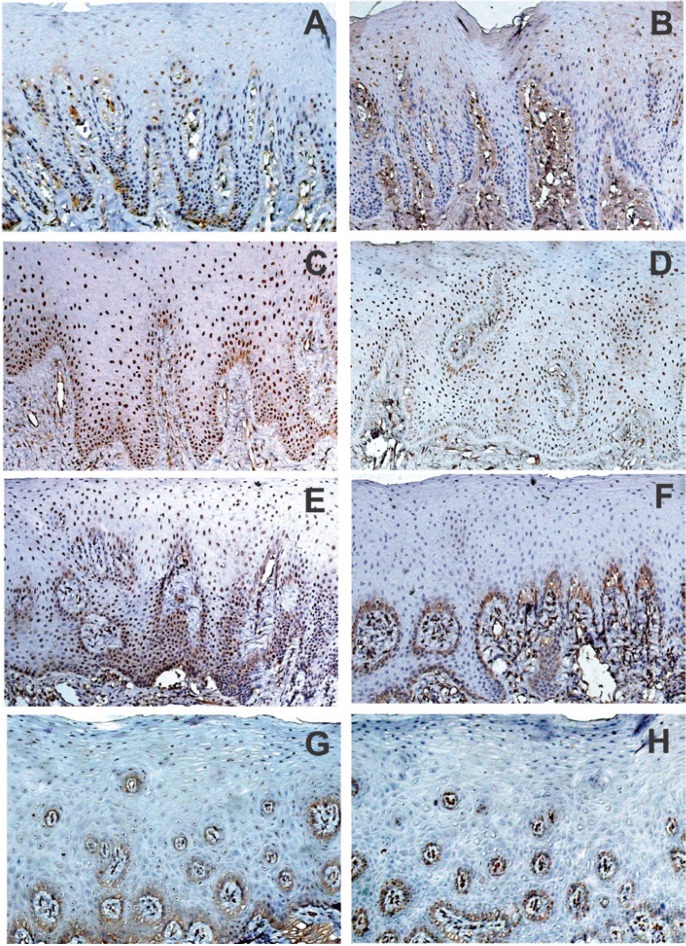
Pattern of α2-integrin and Hsp47 immunohistochemical staining in Family 1 A–B), Family 2 C–D), Family 3 E–F) and control G-H) samples. Note that α2-integrin-positive cells (C and E) were increased in number in Family 2 and Family 3, and Hsp47-positive cells (D) were increased in number in GF Family 2 (original magnification x200).

Nuclear immunoreactivity for α2-integrin and Hsp47 was identified in the basal and suprabasal cell layers of the epithelium (Fig. 2), although mild immunoreactivity was observed for both markers in connective tissue. Hsp47 immunoreactivity was significantly higher in Family 2 compared with NG, GF Family 1 or GF Family 3 (p≤0.05). While the markup to integrin was higher in Family 3 there was no statistically significant difference between the markers for the families studied. The immunohistochemical results of this study are presented in  Table 1.

**Table 1:**
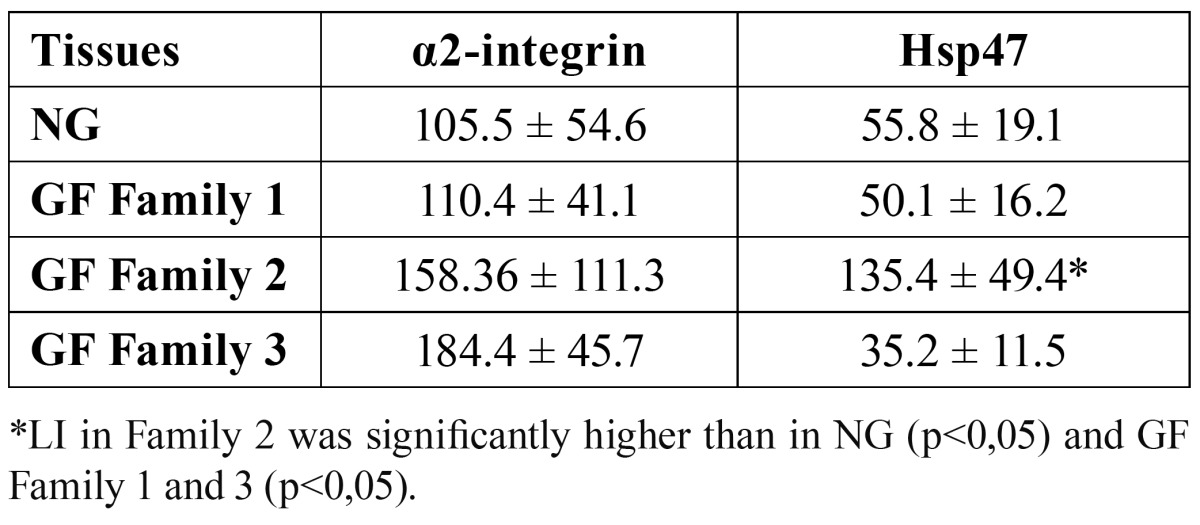
Labeling index (LI) of α2-integrin and Hsp47 in normal gingival and gingival fibromatosis tissues.

## Discussion

Gingival fibromatosis (GF) is the overgrowth of the gingiva characterized by the expansion and accumulation of the connective tissue with the occasional presence of an increased number of cells (1). The excessive accumulation of extracellular matrix (ECM) is the most prominent pathological feature of this disease. In this context the mechanism by which this accumulation of extracellular matrix occurs has been the focus of several studies that have attempted to explain the pathogenesis of various fibrotic diseases, including GF. However, it is not known whether the increased collagen content results from an increase in production by fibroblasts or decreased degradation (15). We examined the expression of the α2-integrin subunit and Hsp47 in gingiva samples of three unrelated Brazilian HGF families, using normal gingiva samples as controls.

Our findings demonstrate integrin expression in the basal and suprabasal layers of epithelial cells of GF as well as discrete expression of this marker in connective tissue, similar to the results of Zhou et al. (15), but unlike the latter study we observed no difference in α2-integrin expression between groups. Moreover, immunostaining for the α2-integrin subunit was mainly in the basal layer of the gingival epithelium. It has been suggested that the increased expression of some integrins in the epithelium could participate in controlling the formation of elongated rete ridges and tissue fibrosis in drug-induced gingival overgrowth (16). Although there was no difference in integrin expression between the groups, our results showed slightly higher expression in GF Families.

The gingiva contains small amounts of collagen type IV compared to collagen I (17); cells expressing α2-integrin preferentially adhere to collagen I (18), the most abundant protein in the extracellular matrix. Research has shown that the invasive behaviour of metastatic C4-2B cells is mediated by the reorganization of lateral α2-integrin subunits and their association and activation via the FAK/src/paxillin/JNK/Rac. This results in increased activation of the matrix metalloproteinases, MMP-2 and MMP-9 (19), which are also present in HGF, although with reduced activity. Thus, the study of this protein is of the most relevance to a better understanding of the aetiology of fibrotic conditions such as GF, and may in future serve as an indicator of changes characteristic of this condition.

Our findings demonstrated Hsp47 expression in the basal and suprabasal layers of epithelial cells of GF as well as discrete expression of this marker in connective tissue. Hsp47 expression was higher in Family 2 in relation to other HGF families and the control group. This finding may be attributed to the clinical, genetic and biological heterogeneity of GFs. This heterogeneity can be illustrated by several examples, such as the study of Sullivan et al. (20), in which genetic sequencing uncovered a mutation in the gene responsible for amelogenesis imperfecta FAM20A associated with GF. Another example is the increased minichromosome maintenance 2 and 5 expression in the epithelium of hereditary gingival fibromatosis associated with dental abnormalities compared with non-syndromic forms of HGF (21). Studies have addressed the use of Hsp47 as a biomarker in cancer (22) and fibrotic diseases (23,24), given the intimate relationship of this protein with the excessive accumulation of collagen, a feature that is present in HGF.

In conclusion, our results confirmed the heterogeneity of GF, since the expression of Hsp47 was higher in only one group. However, future studies are necessary to determine the exact role of the α2-integrin subunit and Hsp47 in the various forms of GF and whether it contributes to GF pathogenesis.

## References

[1] Takagi M, Yamamoto H, Mega H, Hsieh KJ, Shioda S, Enomoto S (1991). Heterogeneity in the gingival fibromatoses. Cancer.

[2] Dongari-Bagtzoglou A (2004). Drug-associated gingival enlargement. J Periodontol.

[3] Coletta RD, Graner E (2006). Hereditary gingival fibromatosis: A systematic review. J Periodontol.

[4] Hakkinen L, Csiszar A (2007). Hereditary gingival fibromatosis: characteristics and novel putative pathogenic mechanisms. J Dent Res.

[5] Singer SL, Goldblatt J, Hallan LA, Winters JC (1993). Hereditary gingival fibromatosis with a recessive mode of inheritance. Case reports. Aust Dent J.

[6] Martelli-Júnior H, Bonan PR, Dos Santos LA, Santos SM, Cavalcanti MG, Coletta RD (2008). Case reports of a new syndrome associating gingival fibromatosis and dental abnormalities in a consanguineous family. J Periodontol.

[7] Ivarsson M, McWhirter A, Black CM, Rubin K (1993). Impaired regulation of collagen pro-α1(I) mRNA and change in pattern of collagen-binding integrins on scleroderma fibroblasts. J Invest Dermatol.

[8] Langholz O, Rockel D, Mauch C, Kozlowska E, Bank I, Krieg T (1995). Collagen and collagenase gene expression in three-dimensional collagen lattices are differentially regulated by α1β1 and α2β1 integrins. J Cell Biol.

[9] Riikonen T, Westermarck J, Koivisto L, Broberg A, Kahari VM, Heino J (1995). Integrin alpha 2 beta 1 is a positive regulator of collagenase (MMP-1) and collagen alpha 1(I) gene expression. J Biol Chem.

[10] Fujimura T, Moriwaki S, Imokawa G, Takema Y (2007). Crucial role of fibroblasts integrins alpha2 and beta1 in maintaining the structural and mechanical properties of the skin. J Dermatol Sci.

[11] Nagata K (1998). Expression and function of heat shock protein 47: a collagen-specific molecular chaperone in the endoplasmic reticulum. Matrix Biol.

[12] Bozzo L, Almeida OP, Scully C, Aldred MJ (1994). Hereditary gingival fibromatosis. Report of an extensive four-generation pedigree. Oral Surg Oral Med Oral Pathol.

[13] Martelli-Júnior H, Lemos DP, Silva CO, Graner E, Coletta RD (2005). Hereditary gingival fibromatosis: report of a five-generation family using cellular proliferation analysis. J Periodontol.

[14] Vigneswaran N, Zhao W, Dassanayake A, Muller S, Miller DM, Zacharias W (2000). Variable expression of cathepsin B and D correlates with highly invasive and metastatic phenotype of oral cancer. Hum Pathol.

[15] Zhou J, Meng LY, Ye XQ, Von der Hoff JW, Bian Z (2009). Increased expression of integrin alpha 2 and abnormal response to TGF-β1 in hereditary gingival fibromatosis. Oral Dis.

[16] Nagata K, Hosokawa N (1996). Regulation and function of collagen-specific molecular chaperone, HSP47. Cell Struct Funct.

[17] Bolcato-Bellemin A L, Elkaim R, Tenenbaum H (2003). Expression of RNAs encoding for α and β integrin subunits in periodontitis and in cyclosporin A gingival overgrowth. J Clin Periodontol.

[18] Kataoka M, Seto H, Wada C, Kido J, Nagata T (2003). Decreased expression of α2 integrin in fibroblasts isolated from cyclosporin A-induced gingival overgrowth in rats. J Periodontal Res.

[19] Slambrouk SV, Jenkins AR, Romero AE, Steelant WFA (2009). Reorganization of the integrin α2 subunit controls cell adhesion and cancer cell invasion in prostate cancer. Int J Oncol.

[20] O'Sullivan J, Bitu CC, Daly SB, Urquhart JE, Barron MJ, Bhaskar SS (2011). Whole-exome sequencing identifies FAM20A mutations as a cause of amelogenesis imperfect and gingival hyperplasia syndrome. Am J Hum Genet.

[21] Martelli-Júnior H, Santos CO, Bonan PR, Moura PF, Bitu CC, León JE (2011). Minichromosome maintenance 2 and 5 expression is increased in the epithelium of hereditary gingival fibromatosis associated with dental abnormalities. Clinics.

[22] Shiuan-Shinn L, Ling-Hsien T, Yi-Ching L, Chung-Hung T, Yu-Chao C (2011). Heat shock protein 47 in oral squamous cell carcinomas and upregulated by arecoline in human oral ephitelial cells. J Oral Pathol Med.

[23] Tagushi T, Nazneen A, Al-Shihri AA, Turkistani KA, Razzaque MS (2011). Heat shock protein 47: A novel biomarker of phenotypically altered collagen-producing cells. Acta Histochem Cytochem.

[24] Totan S, Echo A, Yuksel E (2011). Heat shock proteins modulate keloid formation. Eplasty.

